# Comment on “Acetazolamide Intoxication in an Elderly Patient with Diabetes and Chronic Renal Failure after Cataract Surgery”

**DOI:** 10.1155/2021/9853592

**Published:** 2021-04-02

**Authors:** Michael H. Schwenk

**Affiliations:** Northwell Health, Monter Cancer Center, 450 Lakeville Road, Lake Success NY 11042, USA

In this case report, Kerber and colleagues conclude that hemodialysis is an effective way to remove acetazolamide in patients who have received supratherapeutic dosages and who have severe renal insufficiency [1]. However, I disagree that their case report necessarily demonstrates this conclusion or that previous studies have similarly proved this assertion.

As the authors state, they provide no data documenting acetazolamide serum levels, urinary excretion, spent dialysate concentrations, or dialyzer clearance. Hence, potential other mechanisms were possibly in play to explain the resolution of acetazolamide toxicity.

Since acetazolamide clearance is almost exclusively renal, the patient's kidney function will play a predominant role in its elimination. The patient's serum creatinine was 2.1 mg/dL at baseline, and 4.8 mg/dL at ICU admission, indicating a loss of more than half of the GFR (eGFR by CKD-EPI at baseline was 23 mL/min/1.73 m2 or 27 mL/min/1.73 m2 for nonblack or black, respectively). At discharge 6 days later, the patient's serum creatinine and symptoms had returned to baseline.

Acetazolamide toxicity has been reported in patients while they were receiving chronic hemodialysis therapy and unadjusted acetazolamide dosage regimens [2–4]. The patients' symptoms only resolved 3-6 days after discontinuing the drug. Thus, acetazolamide toxicity can manifest despite dialysis treatments if a marked decrease in daily dosage is not made (e.g., 62.5-125 mg/day).

The study by Vaziri et al. [5] was performed 30 minutes after a single intravenous administration of 500 mg which might not have allowed for complete distribution of the drug, with resultant higher serum concentrations than would be seen postdistribution, and allowing for a greater quantitative removal.

To summarize, this case report does not prove significant acetazolamide clearance by hemodialysis, although the effect of contemporary dialysis therapy on acetazolamide disposition remains to be quantified. It is most likely that stopping drug administration, along with a significant recovery of renal function, is enough to explain this patient's clinical course.

*Michael H. Schwenk*
